# Far From the Biliary Tree: A Case of Overlapping Autoimmune Liver Disease in a Patient Presenting With Sicca Symptoms

**DOI:** 10.7759/cureus.26760

**Published:** 2022-07-11

**Authors:** Keifer Walsh, James Park

**Affiliations:** 1 Family Medicine, Naval Hospital Camp Pendleton, Oceanside, USA

**Keywords:** rheumatic arthritis, hereditary hemochromatosis, sjogren's syndrome, autoimmune hepatitis, primary biliary cholangitis, autoimmune disease

## Abstract

Primary biliary cholangitis (PBC) is a chronic autoimmune condition with many extrahepatic manifestations that are commonly encountered as a patient's primary presenting complaints. Rarely, PBC co-exists as an “overlapping syndrome” with other liver-related autoimmune conditions such as autoimmune hepatitis (AIH). Presented is a rare case of PBC with features of AIH diagnosed in a patient who initially presented with hemoptysis and worsened sicca symptoms due to advanced Sjögren’s syndrome. The patient had a three-year evolution of abnormal liver biochemistry and was found to be a heterozygous carrier for hereditary hemochromatosis (H63D mutation). Given that patients with PBC-AIH are at an increased risk of complications compared to isolated disease from either disorder, early diagnosis and prompt management can help spare patients from cirrhosis, liver failure and transplantation, or even death.

## Introduction

Primary biliary cholangitis (PBC) is an autoimmune liver disease characterized by the progressive destruction of intrahepatic bile ducts. Various genetic and environmental interactions trigger an autoimmune response against biliary epithelial cells, which leads to cholestasis and fibrosis and can ultimately result in liver cirrhosis and failure [1]. The global incidence rate of PBC differs widely among geographic areas, ranging from 40 to 400 per million individuals with a peak incidence in the fifth decade of life and a 10:1 predominance in females compared to males [2]. Symptoms of PBC related to cholestasis typically develop within two to four years of diagnosis and include fatigue, pruritus, dyslipidemia, fat-soluble malabsorption, and osteoporosis, though up to 61% of cases are asymptomatic [2-3]. PBC also has many associations with extrahepatic autoimmune disease, most notably Sjögren’s (up to 73%), thyroid dysfunction (up to 23.6%), cutaneous scleroderma (up to 12.3%), and rheumatoid arthritis (RA) (5.6%) [4-6]. The diagnosis of PBC is made if two of the following three criteria are met: (1) biochemical evidence of cholestasis through the elevation of alkaline phosphatase (ALP) to two times the upper limit of normal, (2) presence of antimitochondrial antibody (AMA) with a titer greater than 1:40, (3) histologic evidence of nonsuppurative destructive cholangitis and interlobular bile duct destruction [7-8].

Rarely, PBC can co-exist as an “overlapping syndrome” with other liver-related autoimmune conditions such as autoimmune hepatitis (AIH). This phenomenon is present in 1-3% of patients with PBC and 7% of patients with AIH [8]. A PBC-AIH overlap syndrome can be diagnosed using the Paris criteria with 92% sensitivity and 97% specificity [9].

For PBC alone, early initiation of ursodeoxycholic acid (UDCA) to slow disease progression is associated with a long-term survival benefit and minimal side effects such as headaches, motility issues, and weight gain. Along with interval monitoring of liver biochemical tests, recommendations for immunizations for hepatitis A and B are given to individuals without serologic evidence of immunity, in addition to abstention from heavy alcohol use. In general, evidence is limited to making treatment recommendations for patients with PBC as well as features of AIH, though it includes UDCA with or without a component of immunosuppression [8].

## Case presentation

A 52-year-old female with a history of Sjögren’s and rheumatoid arthritis presented to the clinic with worsening sicca symptoms and persistent cough with hemoptysis over the past two weeks. She also reported fatigue and arthralgias. She had been seeing a rheumatologist and had been started on azathioprine two years prior, in addition to pilocarpine for dry eyes. She was also taking levothyroxine for hypothyroidism. The patient’s vital signs were all within normal limits. Her physical exam was notable for a non-tender liver edge palpated six centimeters below the mid-clavicular costal margin with negative shifting dullness or fluid wave. Other pertinent negatives included the absence of icteric sclera, skin hyperpigmentation, gland or lymph node prominence or tenderness, focalizing lung findings, active synovitis or contractures, palmar excoriations, or cutaneous vasculitis.

On review of her medical chart, she had an extensive rheumatologic workup notable for positive antinuclear antibody (ANA), positive anti-SS-A with negative anti-SS-B, positive salivary gland biopsy, and positive rheumatoid factor (RF). She also had a history of consistently elevated gamma-glutamyl transferase (GGT) and alanine (ALT) and aspartate (AST) transaminases with normal alkaline phosphatase (ALP) and normal total and direct bilirubin levels. Labs were obtained at her clinic visit and notable for elevated AST 237 IU/L, ALT 223 IU/L, GGT 169 IU/L, and normal ALP 101 IU/L with otherwise normal complete blood count and mildly elevated values on the lipid panel (Table 1).

**Table 1:** Pre-Treatment Laboratory Values Summary of laboratory values for the patient prior to initiating treatment with reference ranges ESR, erythrocyte sedimentation; GGT, gamma-glutamyl transferase; ALT, alanine transaminase; AST, aspartate transaminase; ALP, alkaline phosphatase; TB, total bilirubin; DB, direct bilirubin; HDL, high-density lipoprotein; LDL, low-density lipoprotein; TG, triglyceride

Laboratory	Patient Value	Normal Range
ESR	30 mm/h	0-30 mm/h
GGT	169 IU/L	0-55 IU/L
ALT	223 IU/L	10-45 IU/L
AST	237 IU/L	8-35 IU/L
ALP	101 IU/L	30-120 IU/L
Albumin	4.3 g/dL	3.5-5.0 g/dL
TB	1.2 mg/dL	0-1.2 mg/dL
DB	0.2 mg/dL	0-0.3 mg/dL
Iron	140.0 ug/dL	28-170 ug/dL
Ferritin	389.9 ng/mL	11.0-306.8 ng/mL
HDL	35 mg/dL	≥60 mg/dL
LDL	134 mg/dL	0-130 mg/dL
TG	217 mg/dL	0-150 mg/dL

Based on her autoimmune history and elevated transaminases, an expanded rheumatologic workup was performed, notable for a positive anti-mitochondrial antibody (AMA) 128.6 U (normal < 20.0 U), negative anti-smooth muscle antibody (ASMA) 11.0 U (normal < 19 U), and immunoglobulin A (IgA) 287 mg/dL (normal 87-352 mg/dL). The patient was referred to a gastroenterologist and found to have a normal esophagogastroduodenoscopy. Upon genetic analysis, the patient was noted to be a carrier for hereditary hemochromatosis (heterozygous carrier for H63D mutation). The rest of her workup was negative, including hepatitis panel, alpha-1-antitrypsin, double-stranded DNA and anti-Smith/ribonucleoprotein antibodies, SCL-70 (scleroderma) antibody, and centromere antibody.

**Figure 1:**
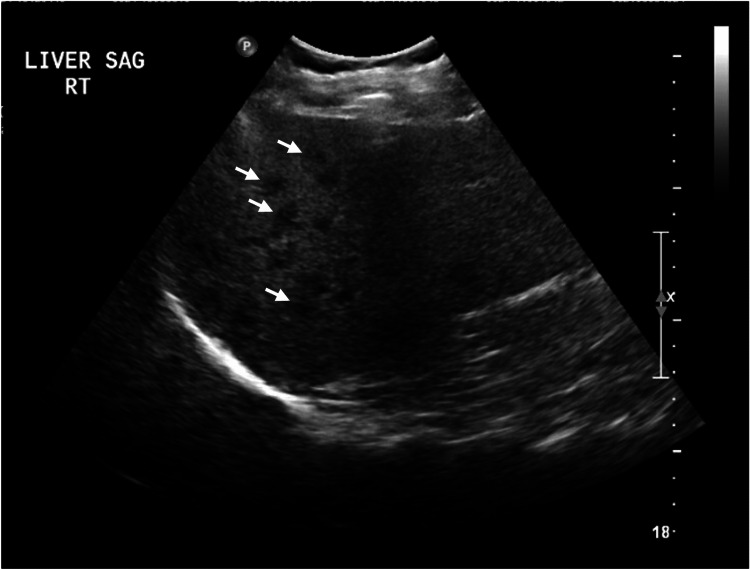
Liver Findings on Ultrasound Imaging Ultrasound demonstrated liver enlargement (17.7 cm) with areas of diffuse hypoechogenicity throughout the liver parenchyma suggestive of fatty infiltration (arrows).

**Figure 2:**
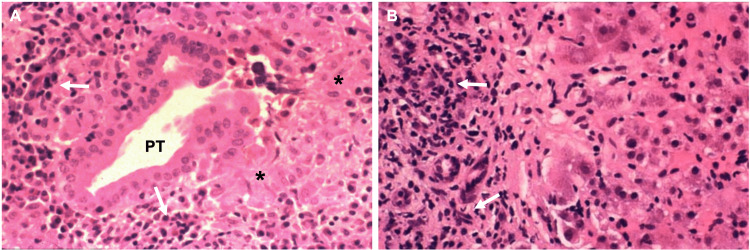
Biopsy Characteristics of Liver Pathology (A) Liver biopsy findings from the patient were consistent with primary biliary cholangitis, including infiltration of lymphocytes (white arrows) surrounding the portal tract (PT) with epithelial cell swelling and hyperplasia (black asterisks). (B) Additionally, evidence of inflammation extended into the lobular parenchyma (white arrows) or “interface activity” that is more characteristic of autoimmune hepatitis.

Imaging with ultrasound demonstrated hepatomegaly with fatty infiltration of the liver (Figure 1). The patient was evaluated by a pulmonologist for her cough and hemoptysis and a CT chest was performed. CT imaging demonstrated prominent mucus and debris along the trachea with subpleural reticulations possibly due to respiratory involvement of her Sjögren’s syndrome (images were unable to be obtained for inclusion in this article). It also noted questionable hepatic surface lobulation that could represent early cirrhosis morphology. With a positive AMA and biochemical pattern of cholestasis and hepatocellular injury, the patient was referred for liver biopsy, which revealed extensive inflammatory infiltrate consisting of lymphocytes and plasma cells surrounding portal tract structures, with the presence of interface hepatitis into lobular parenchyma (Figure 2). No evidence of malignancy was noted.

The patient met the criteria for diagnosis (Table 2) and was started on UDCA at 15 mg/kg. She was also continued on azathioprine at a therapeutic dose of 2 mg/kg for AIH. At the three-month follow-up, the patient had down-trending transaminases and was reporting subjective improvement in symptoms of fatigue and arthralgias.

**Table 2:** Paris Criteria for Diagnosing PBC-AIH Overlap Syndrome Primary hepatic autoimmune conditions may exist as “overlapping syndromes” based on the Paris diagnostic criteria. At least two out of three criteria are required to diagnose each disease (PBC and AIH) or combined to diagnose PBC-AIH syndrome. This patient exhibited the analytical laboratory findings, auto-antibodies, and histopathologic findings (*) of both conditions.

Condition	Laboratory/Histologic Finding
Primary biliary cholangitis (PBC)	1. Alkaline phosphatase (ALP) 1.5 times upper limit of normal (ULN) or gamma-glutamyl transferase (GGT) 5 times ULN
	2. Positive antimitochondrial antibodies (AMA) 1:40*
	3. Liver biopsy with peri-portal inflammatory infiltration*
Autoimmune hepatitis (AIH)	1. Alanine aminotransferase (ALT) 5 times ULN*
	2. Serum immunoglobulin G (IgG) 2 times ULN or positive test anti-smooth muscle antibody (ASMA)
	3. Liver biopsy with moderate or severe periportal or peri-septal lymphocyte activity*

## Discussion

Primary biliary cholangitis (PBC) is a rare but potentially life-threatening autoimmune cholestatic disease of the liver that, when left undiagnosed and untreated, can culminate in end-stage liver cirrhosis. Similar to the patient presented in this case report, PBC has a peak prevalence for those between their fourth and sixth decades of life with a predominance for women [2-3]. Diagnostic criteria for primary hepatic autoimmune diseases rely on biochemical evidence of either cholestasis or hepatocellular damage, presence of auto-antibodies, and histopathological features on liver biopsy [7-9]. While present in fewer than 10% of patients with either PBC or AIH, the “overlapping syndrome” (PBC-AIH) is well-represented in the current case [8]. There are several subtypes and classifications of this condition, though, like other autoimmune disorders, it can be thought to exist on a spectrum of primary tissue involvement. It is hypothesized that patients with underlying bile duct destruction (characteristic of PBC) also possess a genetic predisposition to develop a hepatitic pattern of liver injury (more consistent with AIH), and thus can also be referred to as “PBC, hepatitic form” [10].

The patient presented in this case report met the Paris criteria for PBC-AIH both on histology as well as an elevated ALT and presence of anti-AMA antibody. Though not meeting diagnostic criteria (5 times upper limit of normal (ULN)), she also had an elevated GGT at 3.1 times ULN. Her past medical history of several extrahepatic autoimmune diseases is consistent with other cases of PBC-AIH. PBC is known to have associations with Sjögren’s syndrome in more than half of individuals and to a lesser extent, with thyroid dysfunction, RA, and cutaneous scleroderma [4-6]. Though a majority of patients eventually diagnosed with PBC are asymptomatic, this patient had multiple symptoms on presentation. She presented with cough and hemoptysis along with xerostomia and dry eyes, all potentially attributed to her Sjögren’s diagnosis. She was found to have hepatomegaly both on clinical exam and ultrasound imaging, as well as three years of worsening liver transaminases that prompted an expanded autoimmune work-up.

This case highlights the importance of primary care physicians to not only be familiar with the criteria for autoimmune liver diseases but also to not devalue minor changes in liver biochemistry. Though she had been previously followed by a rheumatologist, the patient had been lost to follow-up and her medical record revealed several inconsistencies regarding the interpretation of her autoimmune laboratory findings between specialists and generalists that she had seen in the past. She had also been noted to have an extensive history of alcohol consumption throughout her medical record. While her AST and ALT abnormalities have been attributed to this, her AST:ALT ratio was inconsistent with this assertion.

Interestingly, this patient was also found to be a heterozygous carrier for hereditary hemochromatosis (HH). On discussion with her gastroenterologist, it is difficult to discern the patient’s elevated ferritin as related to excess collection versus an acute phase reactant given her history of autoimmune disease. In general, HFE H63D heterozygous carriers rarely develop clinically significant iron overload syndromes [11] though may be at increased risk for breast and colorectal cancers [12-13]. As of the time of this publication, there are no reports in the literature regarding increased rates of PBC or AIH in those with the H63D mutation.

Compared to patients with PBC alone, individuals with PBC-AIH have higher rates of portal hypertension, esophageal varices, gastrointestinal bleeding, ascites, and liver failure [14-15]. Studies have demonstrated death or liver transplantation in PBC-AIH at rates twice as high compared to PBC alone at six-year mean follow-up [14] and nearly four times as high compared to AIH alone at the two-year follow-up [16]. At 10 years following diagnosis, 44-48% of patients with PBC-AIH progress to cirrhosis [8,15], and transplant-free survival ranges from 52-92% [8-9,14].

Goals of management for autoimmune liver disease include suppression of the underlying pathogenic process as well as treatment of acute symptoms that result from chronic cholestasis, including pruritus, fatigue, and xerostomia. Evidence is limited regarding specific treatment recommendations for diseases with overlapping characteristics. The 2018 practice guidelines from the American Association for the Study of Liver Diseases (AASLD) guidelines concede that “the clinical benefit and harm of adding immunosuppressive medications require further study,” and recommend tailoring pharmacotherapy to the predominant histologic pattern (PBC or AIH) [17-18]. The 2017 practice guidelines from the European Association for the Study of Liver Diseases (EASL) recommend that in addition to UDCA, immunosuppression be given, or considered, in patients with severe to moderate interface hepatitis, respectively [19]. A meta-analysis for the comparative treatment of various “overlap syndromes” demonstrated that combination therapy with UDCA and immunosuppression may be superior to both UDCA alone and to steroids with or without azathioprine with respect to biochemical improvement and transplant-free survival [8]. The authors concede, however, that these studies are limited by the inclusion of patients with a wide range of histologic severity. Additionally, it has been reported that the degree of baseline interface activity on biopsy (pathognomonic of AIH) is a more accurate predictor of failure with UDCA monotherapy compared to the addition of immunosuppressive therapies [19].

Our patient had already been started on azathioprine for her other rheumatological conditions two years prior to her initial presentation to our clinic. Therefore, given her extrahepatic autoimmune diseases that prompted early immunosuppression, she had theoretically been spared from several years of additional damage due to her PBC-AIH. Relapse rates of AIH up to 90% have been demonstrated when discontinuing immunosuppression. Despite this, a withdrawal trial of immunosuppressives can be considered once remission has been established (normalization of aminotransferases) and maintained for 24 months [19-20]. She was also started on UDCA after confirming the diagnosis with a biopsy and therefore is now on combination UDCA with immunosuppressive therapy. Long-term monitoring for patients with PBC includes liver biochemical and function tests every three to six months in addition to annual screening for thyroid dysfunction and bone mineral densitometry. Of note, our patient did have a degree of long-bone osteopenia on a dual-energy X-ray absorptiometry scan. We also recommended screening for colorectal and breast cancer given her increased risk with HH carrier status.

## Conclusions

Primary liver autoimmune conditions are often associated with extrahepatic manifestations, either as a result of chronic cholestatic symptoms or as distinct, laboratory-identifiable syndromes. Therefore, in patients with a significant rheumatological disease history or when a primary autoimmune workup is being performed in the context of abnormal liver biochemistry, PBC and AIH must both be considered. The Paris diagnostic criteria can be used with a high degree of both sensitivity and specificity to either distinguish or correlate these conditions. Based on the current evidence, early initiation of UDCA with immunosuppressive therapies has been shown to help delay cirrhosis, liver failure and transplantation, and even death.
